# Asynchronous recruitment of low-threshold motor units during repetitive, low-current stimulation of the human tibial nerve

**DOI:** 10.3389/fnhum.2014.01002

**Published:** 2014-12-16

**Authors:** Jesse C. Dean, Joanna M. Clair-Auger, Olle Lagerquist, David F. Collins

**Affiliations:** ^1^Division of Physical Therapy, Medical University of South Carolina, CharlestonSouth Carolina, USA; ^2^Faculty of Physical Education and Recreation and Centre for Neuroscience, University of AlbertaEdmonton, AB, Canada; ^3^Northern Alberta Institute of TechnologyEdmonton, AB, Canada

**Keywords:** motoneuron, electrical stimulation, reflex, sensorimotor integration, motor unit

## Abstract

Motoneurons receive a barrage of inputs from descending and reflex pathways. Much of our understanding about how these inputs are transformed into motor output in humans has come from recordings of single motor units during voluntary contractions. This approach, however, is limited because the input is ill-defined. Herein, we quantify the discharge of soleus motor units in response to well-defined trains of afferent input delivered at physiologically-relevant frequencies. Constant frequency stimulation of the tibial nerve (10–100 Hz for 30 s), below threshold for eliciting M-waves or H-reflexes with a single pulse, recruited motor units in 7/9 subjects. All 25 motor units recruited during stimulation were also recruited during weak (<10% MVC) voluntary contractions. Higher frequencies recruited more units (*n* = 3/25 at 10 Hz; *n* = 25/25 at 100 Hz) at shorter latencies (19.4 ± 9.4 s at 10 Hz; 4.1 ± 4.0 s at 100 Hz) than lower frequencies. When a second unit was recruited, the discharge of the already active unit did not change, suggesting that recruitment was not due to increased synaptic drive. After recruitment, mean discharge rate during stimulation at 20 Hz (7.8 Hz) was lower than during 30 Hz (8.6 Hz) and 40 Hz (8.4 Hz) stimulation. Discharge was largely asynchronous from the stimulus pulses with “time-locked” discharge occurring at an H-reflex latency with only a 24% probability. Motor units continued to discharge after cessation of the stimulation in 89% of trials, although at a lower rate (5.8 Hz) than during the stimulation (7.9 Hz). This work supports the idea that the afferent volley evoked by repetitive stimulation recruits motor units through the integration of synaptic drive and intrinsic properties of motoneurons, resulting in “physiological” recruitment which adheres to Henneman’s size principle and results in relatively low discharge rates and asynchronous firing.

## Introduction

Understanding how motoneurons transform synaptic input into motor output is fundamental to understanding their role in the neural control of human movement. During voluntary contractions, motoneurons receive synaptic inputs from descending and reflex pathways. The present thinking is that the currents that drive motoneuron discharge come from synaptic inputs, intrinsic properties of the neurons themselves (e.g., persistent inward currents; PICs) and metabotropic mechanisms that regulate the strength of these currents over a wide range. These ideas about motoneuron discharge (see Heckman and Enoka, 2012 for review) are based on recordings from motoneurons in reduced animal models (Schwindt and Crill, 1980; Bennett et al., 1998b; Heckman and Lee, 2001), from motor units in humans (Kiehn and Eken, 1997; Gorassini et al., 1998, 2002a) and from computational models (Elbasiouny et al., 2006; Powers et al., 2012). A limitation of studying how motoneurons transform synaptic input into motor output during voluntary contractions in humans is that the temporal characteristics of the synaptic input are inherently ill-defined. One way to circumvent this problem is to study motor unit discharge in response to trains of electrically-evoked afferent impulses. In this way, the temporal characteristics of the synaptic drive are relatively well-defined and the relationship between synaptic drive and motor output can be quantified (Kudina, 1988; Jones and Bawa, 1995; Bawa and Chalmers, 2008; Binboğa et al., 2011). The purpose of the present experiments was to characterize the recruitment and ongoing discharge of human motoneurons when they receive trains of afferent impulses over a range of physiologically relevant frequencies.

Presently, we deliver trains of impulses to human motoneurons by stimulating afferents in the tibial nerve at different frequencies and measure the output by recording the discharge of soleus motor units. The afferent volley evoked by a single suprathreshold pulse delivered to the tibial nerve comprises activity in axons from muscle spindles, Golgi tendon organs and cutaneous receptors (Burke et al., 1983) and traverses mono- and polysynaptic pathways to motoneurons (Burke et al., 1984). The first impulses reach the motor pool in ~15 ms, with impulses traveling along slower axons and/or through polysynaptic pathways arriving ~6–10 ms later (Burke et al., 1984). Although the effects of the afferent volley on motoneurons can be both excitatory and inhibitory (Burke et al., 1983; Marchand-Pauvert et al., 2002; Binboğa et al., 2011), the net result is a relatively synchronous discharge of the motor pool known as an H-reflex (Hoffmann, 1918), which is primarily due to inputs from Ia afferents acting through predominantly monosynaptic pathways (Pierrot-Deseilligny and Mazevet, 2000). During low-intensity repetitive stimulation of the tibial nerve, motor unit activity can develop gradually and has been qualitatively reported to be asynchronous from the stimulation pulses (Lang and Vallbo, 1967; Burke and Schiller, 1976; Collins et al., 2001). Similar contractions develop when vibration is applied over a tendon or muscle belly, a phenomenon known as the tonic vibration reflex (TVR; De Gail et al., 1966; Hagbarth and Eklund, 1966; Burke and Schiller, 1976). In contrast to during electrical stimulation, however, motor unit activity during the TVR has been reported to be phase-locked to the mechanical stimulus (Burke and Schiller, 1976). More recently, we have shown that during repetitive stimulation of the tibial nerve motor unit discharge can be both synchronous with each stimulus pulse, as an H-reflex (Klakowicz et al., 2006; Bergquist et al., 2011; Clair et al., 2011), and “asynchronous” from each stimulus pulse (Collins et al., 2001; Bergquist et al., 2011). The current study represents a quantitative analysis of how such trains of afferent input are transformed into motor output.

We delivered electrical stimulation to the tibial nerve at a low current, below the threshold at which a single pulse elicited a measurable soleus M-wave, H-reflex or ankle extensor torque. Stimulating at such a low current minimized the number of motor units recruited during repetitive stimulation and thus reduced the chances of more than one motor unit discharging simultaneously, at an H-reflex latency, enabling us to more easily quantify motor unit discharge patterns. Low current stimulation also avoided participant discomfort and thus the potential of participants “tensing-up” and/or producing descending commands that contribute to motor unit recruitment. Our working hypothesis is that the recruitment and ongoing discharge of motor units during repetitive electrical stimulation in humans reflects the integration of currents that arise from synaptic drive and intrinsic properties of the neurons themselves, resulting in motor unit discharge that can be either “time-locked” to each stimulus pulse (i.e., H-reflexes) or “asynchronous” from the stimulus pulses. We predicted that low-current electrical stimulation will recruit motor units that are recruited during weak voluntary contractions (Sypert and Munson, 1981), consistent with the synaptic source of recruitment which follows Henneman’s size principle (Henneman et al., 1965; Calancie and Bawa, 1984), and that recruitment will occur over relatively long time periods (on the order of seconds), consistent with the amplification of synaptic inputs by PICs in motoneurons (Spielmann et al., 1993; Bennett et al., 1998a; Heckman and Lee, 2001; Gorassini et al., 2002b). We also predicted that recruitment will occur with no measurable increase in synaptic drive, as quantified by a modified version of the paired motor unit technique. The results of the detailed analyses of motor unit discharge during repetitive stimulation described herein provide insight into how sensory input is transformed into motor output in humans which may prove to be useful for investigating pathophysiological aspects of sensorimotor integration after injury or disease.

## Methods

Experiments were conducted on nine healthy adult volunteers (7 male, 2 female) ranging in age from 22 to 44. All subjects provided written informed consent before participation in the study. The experiments were approved by the University of Alberta Health Research Ethics Board and were conducted in accordance with the Declaration of Helsinki. Low-current electrical stimulation was delivered over the tibial nerve while soleus motor unit activity and ankle extensor torque were recorded. Motor unit recruitment latencies and discharge patterns were compared for a range of stimulation frequencies.

### Experimental setup

To record isometric ankle extensor torque, subjects were seated on the chair of a Biodex dynamometer (System 3, Biodex Medical Systems, Inc, Shirley, NY, USA) with the right hip flexed to 110°, the right knee flexed to 90°, the right ankle at 90°, the right lateral malleolus aligned with the axis of the dynamometer, and the foot strapped to the Biodex footplate. The subject’s trunk was at an approximate angle of 20° reclined from the vertical. Surface EMG was collected using self-adhesive electrodes (1” × 1”, Disposable A10043 Gel Electrodes; Vermed Inc. Bells Falls, VT, USA) placed over the soleus.

Subjects completed 1–3 submaximal contractions of the ankle extensors to warm up prior to the collection of maximum isometric voluntary contraction (MVC) data. They were instructed to push down as if they were pressing a gas pedal, rapidly increase force to a maximum and hold this contraction for 5 s. Following the practice trials, each subject completed between 2 and 4 MVCs, separated by one minute of rest, until the MVC torques varied by less than 10% for two successive contractions. The MVC was quantified as the maximum torque achieved in a single trial during the time period beginning 1 s after the start of the contraction.

After the MVCs were completed, fine wires (0.002 inch diameter, stainless steel; A-M Systems Inc. Carlsborg, WA, USA) were inserted into the soleus muscle belly using a 23-gauge needle to record the activity of single motor units. After insertion, subjects held a weak voluntary contraction (<10% MVC) while the fine wires were slowly retracted until a clear individual motor unit was detected. All EMG data were band-pass filtered between 30 and 5000 Hz. All data were sampled at 10,000 Hz and stored for subsequent analysis.

### Electrical stimulation

Surface electrodes (1” × 1”, Disposable A10043 Gel Electrodes; Vermed Inc. Bells Falls, VT, USA) were placed behind the knee in the popliteal fossa to stimulate the tibial nerve. Rectangular electrical pulses (1 ms duration) were delivered using a Digitimer constant current stimulator (DS7A, Welwyn Garden City, Hertfordshire, England) under computer control. Before each trial, the stimulation current was varied in 0.05 mA increments to find the highest current at which single pulses were sub-threshold for both M-waves and H-reflexes in both the surface and fine wire motor unit EMG and did not produce any detectable ankle extensor torque. Subjects were instructed to remain relaxed during the stimulation, and none reported any discomfort.

Prior to the electrical stimulation trials, subjects performed a voluntary contraction in which they increased ankle extensor torque up to approximately 10% of their MVC, an estimate of the expected maximum torque during the electrical stimulation trials, and then decreased the contraction back to rest. Subjects were instructed to increase and decrease the strength of their contraction as slowly as possible. At the beginning of each electrical stimulation trial, subjects received 3 single pulses of stimulation separated by 5 s to confirm that the stimulation current was sub-threshold. If an M-wave, H-reflex, or torque response was present, the stimulation intensity was reduced and the trial was restarted. Five seconds after the last single pulse, a 30 s stimulation train was delivered at one of seven frequencies: 10, 20, 30, 40, 60, 80, or 100 Hz. The order of these frequencies was randomized for each subject. In many trials soleus EMG activity and ankle extensor torque remained after the stimulation was turned off, in which case subjects were instructed to “relax completely” (>1 s after the end of stimulation), which has been shown to terminate any involuntary sustained activity (Collins et al., 2002). After the seven electrically stimulated trials, one for each frequency, another voluntary contraction with slowly increasing and decreasing torque was performed, with torque increased to the maximum torque level produced during the electrically stimulated trials. The trials for voluntary contractions were performed to determine the torque level at which motor units were first recruited voluntarily and to ensure that the same motor units were recorded during the different electrical stimulation trials. Two minutes of rest separated each trial.

Upon completion of these trials, the fine wire electrodes were slowly retracted while the subjects maintained a weak voluntary contraction. The electrodes were moved until the original motor unit was no longer detectable and a new motor unit was identified. The procedure described in the paragraph above was then repeated. This series of steps was performed until either: (1) the fine wire electrode was pulled out of the muscle belly; (2) four hours had elapsed from the beginning of the experiment; or (3) the subject expressed discomfort from sitting in the same position for the duration of the experiment.

### Motor unit detection

Single motor unit data were analyzed using Spike 2 software (Cambridge Electronic Design Limited; Cambridge, UK). Individual motor units were discriminated using the template matching function of the software and validated by visual inspection. Discriminating individual motor units during electrical stimulation can be complicated by the simultaneous firing of multiple motor units at M-wave or H-reflex latencies, particularly when the stimulation is suprathreshold for M-waves and/or H-reflexes. At the low stimulation currents used for the majority of the trials in this study, motor unit discharge was often unrelated to the timing of each stimulation pulse. This, along with the relatively small number of motor units recruited by the low-current stimulation, allowed easier discrimination of individual motor units.

In the present experiments, we compared the discharge patterns of motor units recruited by a range of stimulation frequencies, and ensured that we were analyzing the same motor unit during successive trials using *post-hoc* template matching. To confirm that the fine wire electrodes did not change location during the electrical stimulation trials, we compared the motor units recruited by a voluntary contraction before these trials to the motor units recruited voluntarily after the electrical stimulation trials. If the same motor units were not activated, the electrical stimulation data were not used.

## Data analysis and statistics

We were interested in the effect of different stimulation frequencies on the recruitment and firing patterns of an individual motor unit. Therefore, we performed detailed analysis only on motor units that were recruited by at least 3 stimulation frequencies; motor units had to be recruited with a stimulation frequency at or below 60 Hz. With this recruitment criterion, we analyzed data collected from a total of 25 distinct motor units.

To address the predictions derived from our working hypothesis, we quantified the following characteristics of motor unit discharge, which are described in more detail in the subsequent paragraphs: (1) ankle extensor torque at recruitment; (2) number of recruited motor units and recruitment latency; (3) temporal relationship between stimulation pulses and the discharge of single motor units; (4) instantaneous discharge rate after electrical stimulation; and (5) discharge rate at the time of recruitment of additional motor units. For each of the statistical tests, *post-hoc* tests were performed as appropriate when significance (*p* < 0.05) was found. *Post-hoc* Tukey comparisons were performed following ANOVAs, and *post-hoc* reduced Chi-square comparisons with a Bonferroni correction were performed following Chi-square tests. All descriptive statistics are reported as mean ± standard deviation.

### Ankle extensor torque at recruitment

We predicted that low-current stimulation would recruit low threshold motor units. To test this, we calculated the ankle extensor torque at the time of motor unit recruitment during both electrically stimulated and voluntary contractions, normalized by the subject’s MVC ankle extensor torque. Motor unit recruitment was defined as the beginning of continuous motor unit firing. In some cases, motor units discharged once or twice, but then went silent again before beginning to fire continuously. Therefore, the beginning of continuous firing was calculated as the first motor unit discharge for which all subsequent interspike intervals were shorter than 600 ms. We used the criteria of 600 ms to identify “continuous” firing as was used previously by Gorassini et al. (2002b) based on the work of Matthews (1996) who identified 300 ms as the longest inter-spike interval for continuously firing soleus motor units. By using a criteria that was double this minimum value we were confident that we were not including in our analyses periods of isolated or sporadic motor unit activity.

### Number of motor units recruited and recruitment latency

Based on our working hypothesis, we expected higher stimulation frequencies to recruit more motor units at shorter latencies. To determine whether stimulation frequency had a significant effect on the number of motor units recruited (out of 25) we performed a Pearson Chi-square analysis. For each trial in which a motor unit was recruited by electrical stimulation, we calculated the recruitment latency as the time between the start of stimulation (i.e., the first stimulus pulse in the train) and the onset of continuous firing as described above. A repeated measures one-way ANOVA was performed to determine whether stimulation frequency had a significant effect on recruitment latency.

### Temporal relationship between stimulation pulses and subsequent discharges

To determine whether motor unit firing was synchronous or asynchronous from the stimulation pulses, we generated post-stimulus time histograms (PSTHs; bin width = 1 ms) of the motor unit discharges following each stimulation pulse for all trials in which the stimulation rate was 10 or 20 Hz. At higher stimulation frequencies, the interval between stimulation pulses was too short (≤33.3 ms) to reliably detect motor units firing at an H-reflex latency (typically ~35 ms). For all trials at 10 and 20 Hz, we calculated the mean and standard deviation of the number of motor unit discharges for each bin of the PSTH. We judged synchronous firing to occur when the number of motor unit discharges at a given latency exceeded the mean plus two times the standard deviation. For all trials, there was a single peak of synchronous firing that was 1–2 ms in duration and occurred between 35 and 44 ms after the stimulation pulse. These peaks were defined to be due to motor units firing at an H-reflex latency. Similar peaks at an M-wave or any other latency were never seen.

We predicted that motor units would fire at a rate within a narrow range, largely asynchronous from the stimulation pulses. The instantaneous discharge rate was calculated for all motor units recruited by 10–40 Hz electrical stimulation. At higher stimulation frequencies more stimulation artifacts were present per unit time and more motor units were recruited, making individual motor units more likely to become obscured. Peak discharge rate was defined as the instantaneous discharge rate values during the 5 s period with the highest average value. We performed repeated measures one-way ANOVAs to identify significant differences in the onset period and the peak discharge rate during 20, 30, and 40 Hz stimulation. These comparisons were performed for the nine motor units that were recruited at each of these stimulation frequencies.

Motor units that fired at an H-reflex latency after a stimulation pulse were assumed to fire as a result of the electrically-evoked afferent input. Therefore, we quantified the time between each stimulation pulse and the previous motor unit discharge, hereby termed “after firing period”, and calculated the probability of a stimulation pulse delivered at this time causing a motor unit to fire at an H-reflex latency. We considered this probability an indicator of motor unit excitability, as a motor unit closer to its firing threshold would be more likely to fire in response to the electrically-evoked afferent volley.

### Instantaneous discharge rate after cessation of electrical stimulation

If, as hypothesized, motor unit discharge was at least partially driven by activation of PICs, we would expect firing to be sustained beyond the end of stimulation. Sustained firing was deemed to be present if a motor unit continued to fire for at least one second after the end of electrical stimulation. To determine whether the electrically-evoked afferent volley influenced motor unit discharge rate, we performed a repeated measures one-way ANOVA to compare the discharge rate during the last second of stimulation to that during the first second of sustained firing after stimulation ended. This comparison was performed for the nine motor units that were recruited at stimulation frequencies of 20, 30, and 40 Hz.

### Discharge at the time of recruitment of additional motor units

Finally, we predicted that motor unit recruitment would occur without an increase in synaptic drive. We tested this using data from trials in which multiple motor units were recruited during the 30 s of electrical stimulation. In a modified version of the “paired motor unit recording” technique (Kiehn and Eken, 1997; Gorassini et al., 1998, 2002a,b), we monitored the discharge rate of the first recruited motor unit (the “control unit”) at the time when an additional motor unit (the “test unit”) was recruited. This approach relies on the assumption that there is a linear relationship between the synaptic drive received by the control and test motor units. If the discharge rate of the control unit was constant, this was taken as an indication that the synaptic drive to the motor pool was also approximately constant. To identify any changes in discharge rate, our surrogate measure of synaptic drive, we performed a repeated measures one-way ANOVA to determine whether the control unit instantaneous discharge rate changed significantly from the 1 s prior to the recruitment of the test unit to the 1 s period following this recruitment. We also tested whether discharge rates were affected by the afferent volley produced by the electrical stimulation by performing a repeated measures one-way ANOVA comparing discharge rate during the last second of stimulation and the first second of sustained firing for both control and test units. These comparisons were performed for the ten instances in which two distinct motor units were recruited by 10, 20, 30, or 40 Hz stimulation.

## Results

**Figure 1:**
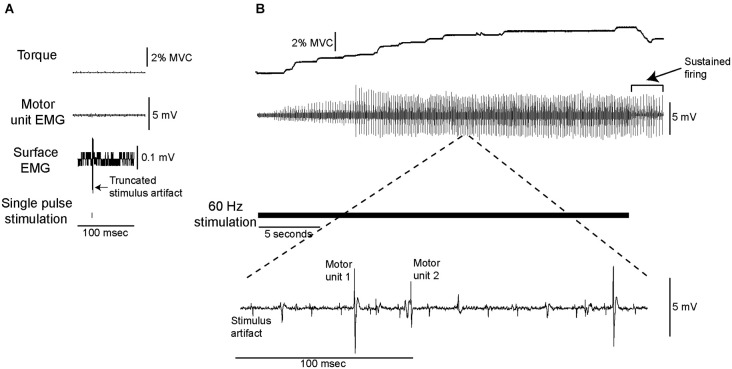
**Data from a single subject showing torque and motor unit activity evoked by low-current electrical stimulation. (A)** The stimulation was delivered at a current at which a single pulse did not elicit any measurable change in ankle extensor torque, surface or intramuscular motor unit EMG. **(B)** Delivering this low-current stimulation for 30 s at 60 Hz (as shown by the solid horizontal line) resulted in the gradual development of motor unit activity and ankle extensor torque.

Electrical stimulation was delivered at a current below the threshold required for a single pulse to elicit either an M-wave or an H-reflex, or produce any ankle extensor torque (Figure 1A). When this stimulation, which ranged from 2.5–10 mA across participants, was delivered at a constant frequency (10–100 Hz), soleus motor unit activity and ankle extensor torque developed gradually in seven of the nine subjects and thus analyses were performed only on data from those seven subjects. Figure 1 shows data from a single subject in whom stimulation at 60 Hz (panel B) resulted in the gradual development of torque (~4% MVC) and the recruitment of 2 distinct motor units whose discharge was sustained after the stimulation ended. Data from 25 motor units that fit our recruitment criterion (see Methods) were analyzed.

### Ankle extensor torque at recruitment

**Figure 2:**
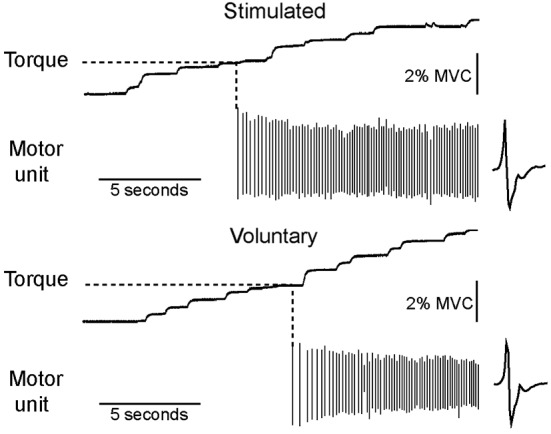
**All motor units recruited during low-current stimulation were also recruited during weak voluntary contractions**. The torque level at which a motor unit first fired was measured for both electrically stimulated and voluntary contractions, as illustrated for a motor unit recorded from a single subject.

Each of the motor units recruited during low-current electrical stimulation was also recruited during a voluntary contraction of similar strength. An example of data recorded from a single subject is shown in Figure 2. All 25 motor units recruited during electrical stimulation were recruited with a relatively weak (<10% MVC) voluntary contraction.

### Number of motor units recruited and recruitment latency

**Figure 3:**
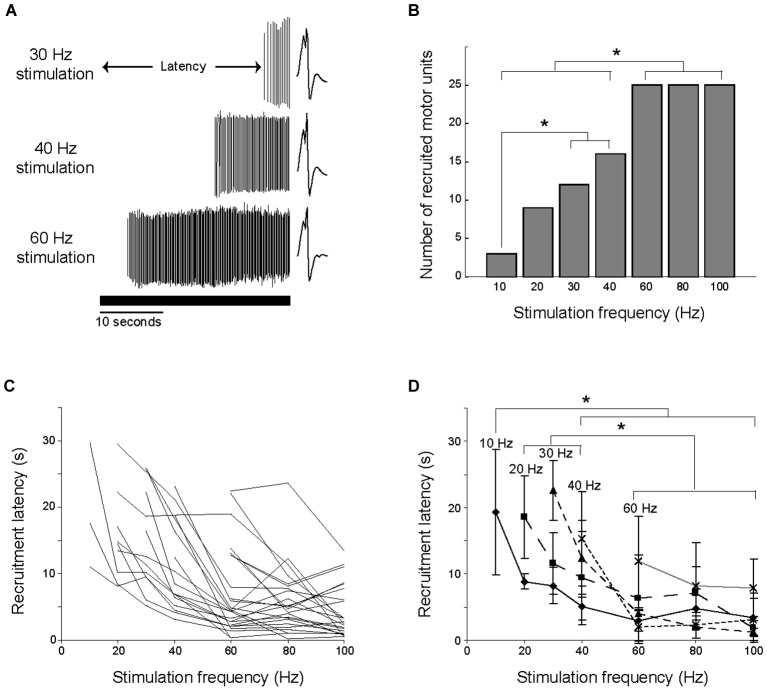
**Number of units recruited and latency of discharge onset were dependent on stimulation frequency. (A)** The time between the beginning of stimulation and the onset of motor unit firing is illustrated for a single motor unit at three stimulation frequencies. The solid horizontal line shows the duration of the stimulation. **(B)** Higher stimulation frequencies recruited more motor units, with significant differences (*p < 0.05) between the indicated stimulation frequencies. **(C)** The change in recruitment latency with stimulation frequency is illustrated for each of the 25 motor units. The minimal stimulation frequency required to recruit each motor unit varied. **(D)** The 25 motor units were grouped by the minimal stimulation frequency required for their recruitment, and the average recruitment latencies were calculated for each group. Higher stimulation frequencies recruited motor units at shorter latencies, with significant differences (*p < 0.05) between the indicated stimulation frequencies.

During 30 s of constant frequency low-current stimulation, motor units did not respond to the first stimulation pulse, but rather were silent for a given latency before being recruited (Figure 3A), or were not recruited at all. Higher stimulation frequencies recruited significantly more motor units within the 30 s of stimulation (Chi-square, *p* < 0.05; Figure 3B). A motor unit recruited by a given stimulation frequency was always recruited by each of the higher stimulation frequencies. For example, the 3 motor units recruited at 10 Hz stimulation were also recruited at all of the other stimulation frequencies.

Recruitment latencies spanned almost the entire course of the electrical stimulation, ranging from 0.5 to 29.6 s (Figure 3C). Several of the motor units were not recruited for a relatively long time, as 22 of the motor units had a recruitment latency longer than 10 s in at least one trial, and 9 of the motor units had a recruitment latency longer than 20 s in at least one trial. Higher stimulation frequencies recruited motor units at significantly shorter latencies than low frequencies (ANOVA, *p* < 0.05). This is illustrated in Figure 3D, in which the 25 motor units are grouped into populations based on the minimum stimulation frequency at which they were recruited. For example, the line labeled 10 Hz illustrates the average recruitment latency across stimulation frequencies for the 3 motor units recruited during 10 Hz stimulation. The effect of increasing stimulation frequency on decreasing recruitment latency is evident in each of these populations.

### Temporal relationship between stimulation pulses and subsequent discharges

**Figure 4:**
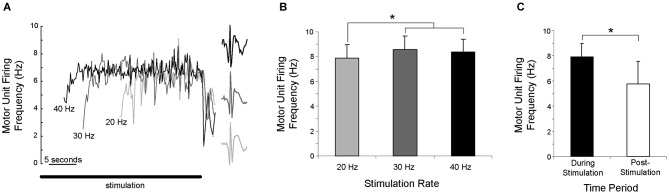
**Motor unit discharge rate was not proportional to stimulation frequency. (A)** As illustrated for a single motor unit activated by three stimulation frequencies, there is a gradual increase in discharge rate, which then reaches a steady-state. The solid horizontal line shows the duration of the stimulation. **(B)** Averaged across subjects, the peak discharge rate was significantly (*p < 0.05) lower during 20 Hz stimulation than during 30 or 40 Hz stimulation. **(C)** Averaged across subjects and stimulation frequency, motor unit discharge rate during the first second of sustained firing was significantly (*p < 0.05) lower than the discharge rate during the last second of stimulation.

Once motor units were recruited, their discharge rate increased until a steady-state was reached, as shown for a single motor unit in Figure 4A. Motor unit discharge rate was not proportional to stimulation frequency, as the discharge rate reached approximately the same peak level regardless of stimulation frequency. Peak motor unit discharge rates were compared for the 9 motor units that were recruited during 20, 30 and 40 Hz stimulation. Peak discharge rate was significantly lower, albeit only slightly, with a stimulation frequency of 20 Hz (motor unit discharge rate = 7.8 ± 1.1 Hz) than with stimulation frequencies of 30 Hz (8.6 ± 1.1 Hz) or 40 Hz (8.4 ± 1.1 Hz), which were not significantly different (Figure 4B).

**Figure 5:**
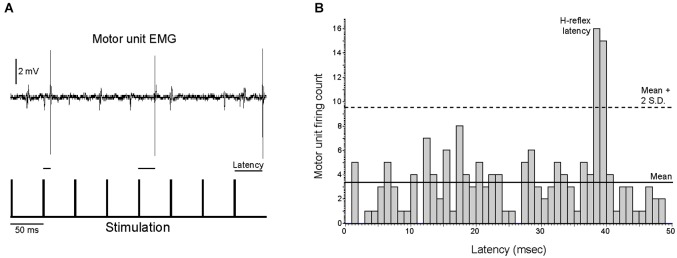
**Motor unit activity is only partially asynchronous from the stimulation pulses. (A)** The latencies between motor unit discharges and the previous stimulation pulses were quantified for the nine motor units that were recruited with a stimulation frequency of ≤20 Hz, as illustrated for an excerpt of activity for a single motor unit. **(B)** Motor units fired at an H-reflex latency more often than would be expected from random chance, as illustrated for a single motor unit that fired at an H-reflex latency 18% of the time. For all analyzed motor units, this H-reflex latency peak exceeded the mean plus two standard deviations of the number of discharges per bin.

Qualitatively, motor units appeared to fire asynchronously from the stimulation pulses, as the latency between stimulus pulses and motor unit action potentials was not constant (Figure 5A, see also Figure 1B). PSTH analysis revealed that motor units fired at an H-reflex latency (1–2 ms peak ~35–44 ms after a stimulation pulse) more often than would be expected from a completely asynchronous distribution, as illustrated in Figure 5B for a single motor unit. This analysis was performed for all trials in which a motor unit was recruited by a stimulation frequency of 10 or 20 Hz. In these 12 trials (*n* = 9 motor units), 24% (range 18–58%) of the motor unit discharges occurred at an H-reflex latency after a stimulation pulse, compared to the ~2–4% of the time that would indicate true asynchronous firing.

**Figure 6:**
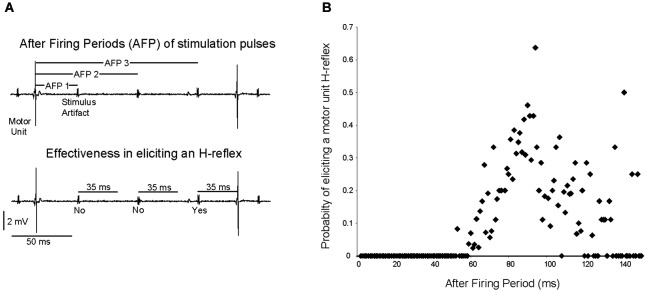
**The probability of a stimulation pulse eliciting an H-reflex depends on the time since the motor unit last fired. (A)** As shown for a single motor unit, the time since the previous motor unit discharge was measured for each stimulation pulse, and termed the “after firing period”. It was then determined whether this stimulation pulse elicited an H-reflex. **(B)** Across subjects, the probability of a stimulation pulse eliciting a motor unit H-reflex was calculated for each 1 ms after firing period increment.

The probability of a stimulation pulse causing a motor unit to fire at an H-reflex latency was dependent upon the timing of the pulse. The “after firing period”, the time between the stimulation pulse and the previous motor unit discharge, was calculated for all motor units recruited by 10 or 20 Hz stimulation (12 trials, 9 motor units), as illustrated for a single motor unit in Figure 6A. Averaged across motor units, stimulation pulses delivered within 50 ms after a motor unit discharge (after firing period <50 ms) never caused the motor unit to fire at an H-reflex latency (Figure 6B). As the after firing period grew longer, the probability of the motor unit firing at an H-reflex latency after a stimulation pulse increased up to a maximum of 0.63 with an after firing period of 92 ms. Again, this analysis was performed for the 12 trials in which a motor unit was recruited by a stimulation frequency of 10 or 20 Hz.

### Instantaneous discharge rate after cessation of electrical stimulation

Motor units often continued to fire after stimulation had ended, with sustained firing for at least one second occurring in 102 of 115 trials (89%). For the 9 motor units recruited during 20, 30 and 40 Hz stimulation, the discharge rate during the last second of stimulation (7.9 ± 1.1 Hz) was significantly higher than the discharge rate during the first second of sustained firing (5.8 ± 1.8 Hz) after stimulation ended (Figure 4C). The duration of the sustained activity was not quantified, as subjects were instructed to relax completely in order to allow 2 min of rest between trials and limit experiment duration. Although subjects stated that they were relaxed and were not voluntarily contracting, instructing them to “relax completely” terminated the motor unit activity.

### Discharge at the time of recruitment of additional motor units

**Figure 7:**
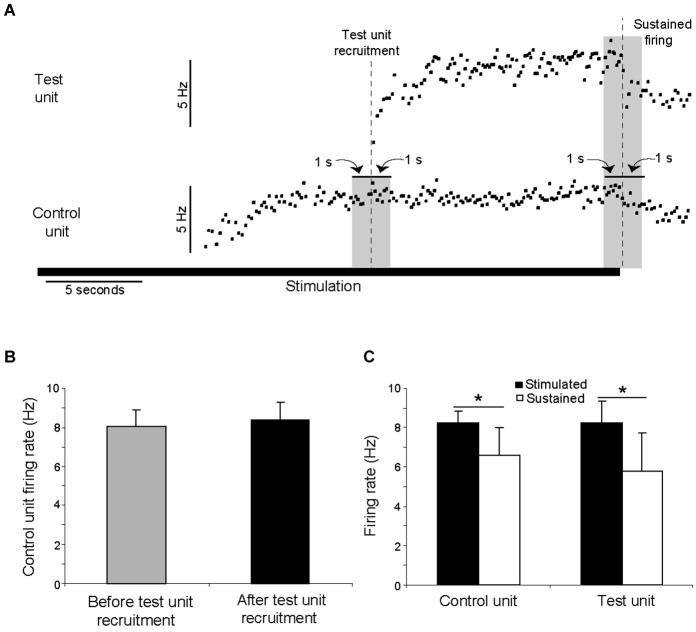
**The recruitment of additional motor units during electrical stimulation was not associated with an increase in the discharge rate of already active motor units. (A)** A motor unit (control unit) was recruited by electrical stimulation, followed after approximately 8 s by the recruitment of an additional (test) motor unit. The control unit discharge rate did not increase when the test unit was recruited. These data are from a single trial in which the stimulation was delivered at 20 Hz for 30 s. The duration of the stimulation is shown by the solid horizontal line. The discharge rates of both units decreased when stimulation ended. **(B)** In ten instances, a control unit fired at its steady-state frequency for at least one second before a test unit was recruited. The discharge rate of the control unit during the 1 s period prior to the recruitment of the test unit was not significantly different than the discharge rate during subsequent 1 s period (*p* > 0.05). **(C)** For the same ten instances, the discharge rate of both the control and test unit decreased significantly (*p* < 0.05) once the stimulation ended.

In 10 trials, two clearly distinguishable motor units were recruited during the stimulation, as illustrated in Figure 7A. The discharge rate of the first motor unit recruited (“control unit”) followed the pattern described above, with an initial increase followed by a steady-state in discharge rate. This discharge rate then remained constant even as additional motor units (“test units”) were subsequently recruited. In no trials did the discharge rate of a control unit increase as a test unit was recruited. Across the ten analyzed paired motor unit recordings, the control unit discharge rate during the 1 s period immediately prior to the recruitment of the test unit was not significantly different than the control unit discharge rate during the subsequent 1 s period (ANOVA, *p* > 0.05; Figure 7B). The discharge rates of both the control and test units decreased significantly once the stimulation ended (ANOVA, *p* < 0.05; Figure 7C).

## Discussion

Repetitive electrical stimulation of the tibial nerve, delivered at an intensity that was below threshold for producing H-reflexes (or M-waves) with a single pulse, resulted in the gradual recruitment of low threshold soleus motor units. A wide range of relatively high electrical stimulation frequencies resulted in a narrow range of relatively low motor unit discharge frequencies. The electrically-evoked afferent volley influenced motoneuron discharge “directly”, resulting in motor unit discharge that was time-locked to the stimulus pulses as H-reflexes, and “indirectly”, as reflected by motor unit discharge that was temporally uncoupled from the stimulation. Accordingly, the discharge of recruited motor units was consistent with the physiological activity that occurs during voluntary contractions, including recruitment according to Henneman’s size principle, relatively low discharge rates and asynchronous firing.

### Electrical stimulation recruited low threshold motor units

Electrical stimulation of afferents in the tibial nerve recruited a population of motor units with a low threshold for voluntary activation. Each of the 25 motor units recruited by the electrically-evoked afferent volley was also recruited during a weak voluntary contraction, indicating that this method of activating motor units follows Henneman’s size principle as has been shown previously (Henneman et al., 1965; Calancie and Bawa, 1984). This contrasts with the random recruitment order that occurs when motor units are recruited as M-waves by the depolarization of motor axons when the stimulation is delivered at higher current stimulation than was used presently (Bickel et al., 2011). These differences in how motor units are recruited by “central” and “peripheral” pathways has implications for using electrical stimulation to generate contractions for rehabilitation (see Implications, below).

### Stimulation efficacy increased over time

The effectiveness of the stimulation in recruiting motor units increased during the 30 s stimulus trains. Motor units were recruited at latencies ranging from 0.5 to 29.6 s, with the upper bound limited by stimulation duration. Recruitment latencies were longest when stimulation frequencies were lowest (10–40 Hz). Temporal summation of EPSPs does not occur when excitatory volleys are greater than 50 ms apart (frequencies <20 Hz) in either reduced animal models (Curtis and Eccles, 1960) or humans (Táboríková and Sax, 1969; Pierrot-Deseilligny et al., 1976; Ashby and Zilm, 1982; Powers and Turker, 2010). Therefore, the reported long recruitment latencies, particularly during stimulation at 10 Hz, cannot be caused solely by temporal summation of synaptic inputs, but must require an increase in either synaptic drive or excitability of the motor pool. These relatively long recruitment latencies are similar to previously reported motor unit recruitment with vibration (Kiehn and Eken, 1997), electrical stimulation of a peripheral nerve (Lang and Vallbo, 1967), extracellular activation (Spielmann et al., 1993) and intracellular current injection (Heckman and Lee, 2001).

### Motor units discharged “asynchronously” and at relatively low frequencies

Motor units did not discharge one-to-one with the stimulation frequency, but remained within a relatively narrow range (7.8–8.6 Hz) as stimulation frequency doubled from 20 to 40 Hz. This finding contradicts the qualitative results of Lang and Vallbo (1967), who reported that the mean firing of a motor unit increased as stimulation frequency increased, but is consistent with the “preferred” firing range of motor units, in which discharge rate does not scale linearly with synaptic input (Hornby et al., 2002). Motor units continued to discharge even after electrical stimulation ended, albeit at a significantly slower rate, indicating that afferent drive contributed to motor unit discharge, but was not solely responsible for the observed firing rate.

Motor unit discharge was largely, but not completely, asynchronous from the stimulation pulses, providing more evidence that discharge was influenced both directly, by the afferent volley (i.e., H-reflexes), and indirectly, by mechanisms intrinsic to the motoneurons themselves (i.e., asynchronous firing). The majority (76 %) of motor unit discharges did not occur at an M-wave or H-reflex latency after a stimulation pulse, eliminating an increase in neurotransmitter release associated with post-tetanic potentiation (Lloyd, 1949; Hultborn et al., 1996), or activity dependent increases in either motor or sensory axon responsiveness to electrical stimulation following repeated pulses (Burke et al., 2001) as possible explanations for the gradual development of motor unit activity. Conversely, if motor unit firing was not influenced by the afferent volley, motor unit discharge would be completely asynchronous from the stimulation, as previously reported qualitatively (Lang and Vallbo, 1967; Burke and Schiller, 1976; Collins et al., 2001). The present quantitative results demonstrate that when appropriately timed (at least 50 ms after the previous motor unit discharge), the electrically-evoked afferent volley was often sufficient to cause motor units to discharge. As the time since the last discharge increased, the probability of a stimulation pulse causing a response at an H-reflex latency increased, up to a maximum of 0.63 for pulses delivered 92 ms after a prior discharge. A similar recovery time course was found for soleus motor units during voluntary contractions (Jones and Bawa, 1995); this recovery during voluntary contractions depended on motor unit firing rates and was consistent with the predicted membrane voltage trajectories during the interspike interval.

### Possible mechanisms

The amplification of synaptic input by PICs in spinal neurons (Lee and Heckman, 2000; Heckman and Lee, 2001; Heckman and Enoka, 2012) or the neuromodulatory facilitation of PICs (Perrier et al., 2002) could produce the type of motor unit discharge seen in the present experiments. Consistent with this idea, the paired motor unit recordings provide evidence that the gradual recruitment presently observed was not due to an increase in synaptic drive but rather was due to an increase in excitability of the motor pool. This technique has been used previously to monitor synaptic drive to the motor pool at a time when new motor units are being recruited (Kiehn and Eken, 1997; Gorassini et al., 1998, 2002a,b). In the present study, control motor units were sensitive to changes in synaptic drive; they fired at approximately 8 Hz, well below the maximal discharge rate of 20 Hz of voluntarily recruited soleus motor units (Bellemare et al., 1983). Additionally, motor unit discharge rates increased significantly when stimulation frequency increased from 20 to 30 Hz and discharge rates of both control and test units decreased significantly when stimulation ended. Thus, although “control” units were sensitive to changes in the electrically-evoked afferent volley, their discharge rate did not increase significantly at the time of recruitment of “test” units. This result suggests that recruitment of the additional motor unit was due to an increase in current provided by a post-synaptic mechanism such as activation of PICs in the motoneuron, not a pre-synaptic mechanism such as increased synaptic drive (Tokuno et al., 2003). We acknowledge that the control unit discharge rate may not exactly reflect changes in synaptic drive (Fuglevand et al., 2006), particularly at higher stimulation frequencies. However, previous paired motor unit results identify motoneurons as the most likely location of PIC activation (Powers et al., 2008; Vandenberk and Kalmar, 2014).

Increases in descending inputs or the magnitude of the electrically-evoked afferent volley cannot be ruled out as having influenced the observed motor unit recruitment. For example, ascending input to the brainstem may have prompted the release of monoamines such as serotonin from the raphe nucleus (Alvarez et al., 1998) or norepinephrine from the locus coeruleus (Lai et al., 1989). To directly address this possibility, the experiments described herein should be repeated in patients with complete spinal cord injuries, in whom the electrical stimulation of peripheral nerves would not be expected to have supraspinal effects. While our paired motor unit recordings suggest that it is unlikely that increased synaptic drive to the motor pool contributed to the observed contractions, we cannot rule out the possibility that increases in the descending input or the electrically-evoked afferent volley contributed to motor unit recruitment but was too small to significantly increase the discharge of our control unit. However, similar gradually-developing contractions and sustained firing have been observed in individuals with complete spinal cord injuries (Nickolls et al., 2004), suggesting that descending drive is not the primary contributor to the motor unit discharge behavior described in the present experiments. Further, repetitive activation of axons typically results in an activity-dependent hyperpolarisation, thus increasing axonal thresholds to electrical stimulation, which may have reduced and not increased the magnitude of the electrically-evoked afferent volley over the course of a stimulus train.

### Implications

The present results describe how motoneurons transform afferent feedback into motor output in individuals with no neurological impairments. While such transformation may be a key contributor to voluntary contractions, afferent feedback during voluntary movements would be more temporally diffuse than the relatively synchronous activation of sensory axons that occurs during electrical stimulation. The synchronous nature of the electrically-evoked afferent volley is a limitation of the present study as it decreases the physiological relevance of our findings, however, it is a strength in that it allowed us to characterize how motoneurons respond to discrete excitatory volleys which would not be possible with vibration or voluntary contractions. We suggest that utilizing this “low-current stimulation” approach on individuals with neurological impairments may provide novel insight into how sensorimotor transformation is affected by injury or disease.

These findings also have implications for understanding how motor units are recruited during neuromuscular electrical stimulation. Electrical stimulation is used for rehabilitation after an injury or disease to prevent muscle atrophy, generate functional movements, or preserve motor unit types. Electrical stimulation, however, recruits motor units in a non-physiological order (Sheffler and Chae, 2007; Bickel et al., 2011) and leaves slow fatigue-resistant motor units, which are the most likely to develop disuse atrophy, (Burnham et al., 1997), relatively inactive. In contrast, generating muscle contractions through afferent feedback recruits motor units synaptically (Henneman et al., 1965; Bennett et al., 1998a), thereby first activating the fatigue-resistant muscle fibers (Sypert and Munson, 1981) with relatively weak stimulation. Thus, electrical stimulation delivered to recruit motor units by the electrically-evoked afferent volley may prove to be beneficial to reduce muscle atrophy and generate fatigue-resistant contractions. Such synaptic recruitment could also help maintain the biophysical properties of motoneurons, which are sensitive to changes in synaptic drive (see Gardiner et al., 2005 for review). In addition, modulating the excitability of a motor pool with low-current electrical stimulation of peripheral nerves may facilitate residual descending drive to augment voluntary commands and generate muscle contractions.

### Conclusions

Presently we show that human motoneurons transform a wide range of synaptic input frequencies into a relatively narrow range of low output frequencies. Motor unit discharge was strongly influenced by properties intrinsic to the motoneurons themselves and was not solely driven by processes temporally-coupled with the synaptic input. This work supports the idea that sensory input evoked during electrical stimulation activates PICs in motoneurons, and describes a method of studying sensorimotor integration in humans with a more clearly defined input to spinal neurons than those produced by vibration or voluntary contraction.

## Conflict of interest statement

The authors declare that the research was conducted in the absence of any commercial or financial relationships that could be construed as a potential conflict of interest.
